# Notes and News

**Published:** 1904-04-30

**Authors:** 


### Notes and News.

Prince Francis of Teck has accepted the Presidency of the Middlesex Hospital in place of the late Duke of Cambridge.

Up to the present time upwards of 160 cases of plague have been reported in the Transvaal with about 77 deaths, amongst the latter number being 8 white patients.

The annual meeting of the National Refuges for Homeless and Destitute Children will be held on Friday, April 29, at 3 p.m., in Exeter Hall. The Earl of Jersey will preside.

At the last quarterly meeting of the Royal College of Surgeons, the Jacksonian Prize was awarded to Mr. Stephen Mayou, F.R.C.S., and the Triennial Prize to Mr. Rupert Bucknall, F.R.C.S.

No great belief in the efficacy of a residence in an inebriates' home as a cure for drunkenness was expressed recently at the North London Police Court, where a magistrate sent a woman of no fixed home to an inebriates' retreat. He remarked in nine out of ten cases the ratepayers' money was thrown away in maintaining drunken women in comparative luxury. The missionary present also made unfavourable remarks upon the institutional treatment of inebriates

The Lord Hugh Cecil, M.P., President of the Greenwich District of the League of Mercy, which is an auxiliary founded by Royal Charter to King Edward's Hospital Fund, for the collection of small sums of money for the hospitals, presided last week at a drawing-room meeting held at 6 Vanbrugh Terrace, Blackheath, S.E., by the kindness of Miss Cecilia E. Stone, Mayoress of Greenwich and Lady President of the District. There was a large and enthusiastic audience. The meeting was addressed by the Lord Hugh Cecil, M.P., Dr. Herbert J. Paterson, (a Vice-President of the East Marylebone District), the Rev. S. Martyn Bardsley, Vicar and Rural Dean of Greenwich, and Captain Walter Vernon Anson, R.N.

Mr. J. Rockefeller has given 500,000 dollars to the Johns Hopkins Hospital on account of the losses which it sustained by the fire which took place in Baltimore.

A meeting of the St. John's Hospital for Diseases of the Skin, Leicester Square, will be held on May 5, at 4.30, under the presidency of the Earl of Chesterfield, for the purpose of passing resolutions for the incorporation of the hospital.

The foundation-stone of a Cottage Hospital has been laid at Ilkley. The building is to provide initial accommodation for six patients, means of extension being secured. The estimated cost is £2,250. The hospital will serve as a Coronation memorial.

Mr. Henry Stedall, of the Firs, Hampstead Heath, N.W., has been elected Chairman of the Committee of Management of the Mount Vernon Hospital for Consumption and Diseases of the Chest, Hampstead and Northwood, Middlesex.

The Bishop of Croydon has promised to speak at Exeter Hall on Friday afternoon, 29th inst., on the occasion of the sixty-first annual meeting of the National Refuges and Training Ships *Arethusa* and *Chichester*, when the Earl of Jersey, President, will take the chair, and a choir of children and sailor boys will sing. Tickets may be obtained of the Secretary, Mr. H. Bristow Wallen, 164 Shaftesbury Avenue, W.C.

Mr. George Herring, who has done so much for the London hospitals, and for the North-West London Hospital in particular, is appealing for funds for this institution. Regular annual subscribers are more particularly desired, as out of an expenditure of £4,500 per annum only £600 can be counted upon from this source. The hospital has done good work since 1878, and it serves the densely and poorly populated district of Kentish Town.

The late Mr. J. E. Cook, of Cobham, has bequeathed as legacies in reversion £1,000 to the Metropolitan Convalescent Institution, £2,000 to the London Hospital, with a present annuity of £60, £200 to St. Thomas's Hospital, £100 to the Bolingbroke Hospital, £300 to All Saints' Convalescent Home, Eastbourne, £200 to the Seaford Convalescent Home, and £200 to the Sea Bathing Hospital, Margate.—The late Mr. Edmund Mansbridge, of Portsmouth, has bequeathed £1,000 to the Royal Portsmouth Hospital, and £500 to the Portsmouth and South Hants Eye and Ear Infirmary.—The Royal Sea-Bathing Hospital will be the fortunate recipient of about £45,000 through the will of the late Mr. Charles Alfred Swinbourne, of Andover. Mr. Swinbourne was a vice president of the hospital. —Mr. Edward Davis, of Cheltenham, has made a donation of £1,000 to the London Hospital.—The Royal Free Hospital, the Children's Hospital, Great Ormond Street, St. Thomas's Hospital, and the Chelsea Hospital for Women, will benefit by the will of the late Mr. John Forbes, K.C., who has bequeathed the ultimate residue of his estate to them.

